# Meningococcal Vaccine for Hajj Pilgrims: Compliance, Predictors, and Barriers

**DOI:** 10.3390/tropicalmed4040127

**Published:** 2019-10-15

**Authors:** Al-Mamoon Badahdah, Fatimah Alghabban, Wajd Falemban, Abdullah Albishri, Gouri Rani Banik, Tariq Alhawassi, Hatem Abuelizz, Marwan A. Bakarman, Ameneh Khatami, Robert Booy, Harunor Rashid

**Affiliations:** 1 National Centre for Immunisation Research and Surveillance (NCIRS), The Children’s Hospital at Westmead, Westmead, NSW 2145, Australiarobert.booy@health.nsw.gov.au (R.B.); harunor.rashid@health.nsw.gov.au (H.R.); 2 The Discipline of Child and Adolescent Health, The Children’s Hospital Westmead Clinical School, Faculty of Medicine and Health, The University of Sydney, Westmead, NSW 2145, Australia; ameneh.khatami@gmail.com; 3 Department of Family and Community Medicine, Faculty of Medicine in Rabigh, King Abdulaziz University, Jeddah 22252, Saudi Arabia; dr.amb4@gmail.com (A.A.); bakarmanm@gmail.com (M.A.B.); 4 Faculty of medicine, Umm Al-Qura University, Makkah 24381, Saudi Arabia; ghabban.1993@gmail.com (F.A.); W.f-94@hotmail.com (W.F.); 5 Department of Clinical Pharmacy, College of Pharmacy, King Saud University, Riyadh 12372, Saudi Arabia; tarriq@ksu.edu.sa (T.A.); habuelizz@ksu.edu.sa (H.A.); 6 Department of Infectious Diseases and Microbiology, The Children’s Hospital at Westmead, Westmead, NSW 2145, Australia; 7 Marie Bashir Institute for Infectious Diseases and Biosecurity, School of Biological Sciences and Sydney Medical School, University of Sydney, Westmead, NSW 2145, Australia

**Keywords:** Hajj, meningococcal disease, vaccine uptake, pre-travel health advice

## Abstract

Background: Major intercontinental outbreaks of invasive meningococcal disease associated with the Hajj occurred in 1987, 2000, and 2001. Mandatory meningococcal vaccination for all pilgrims against serogroups A and C and, subsequently, A, C, W, and Y controlled the epidemics. Overseas pilgrims show excellent adherence to the policy; however, vaccine uptake among domestic pilgrims is suboptimal. This survey aimed to evaluate meningococcal vaccine uptake among Hajj pilgrims and to identify key factors affecting this. Methods: An anonymous cross-sectional survey was conducted among pilgrims in Greater Makkah during the Hajj in 2017–2018. Data on socio-demographic characteristics, vaccination status, cost of vaccination, and reasons behind non-receipt of the vaccine were collected. Results: A total of 509 respondents aged 13 to 82 (median 33.8) years participated in the survey: 86% male, 85% domestic pilgrims. Only 389/476 (81.7%) confirmed their meningococcal vaccination status; 64 individuals (13.4%), all domestic pilgrims, did not receive the vaccine, and 23 (4.8%) were unsure. Among overseas pilgrims, 93.5% certainly received the vaccine (6.5% were unsure) compared to 80.9% of domestic pilgrims (*p* < 0.01). Being employed and having a tertiary qualification were significant predictors of vaccination adherence (odds ratio (OR) = 2.2, 95% confidence interval (CI) = 1.3–3.8, *p* < 0.01; and OR = 1.7, CI = 1–2.5, *p* < 0.05, respectively). Those who obtained pre-Hajj health advice were more than three times as likely to be vaccinated than those who did not (OR = 3.3, CI = 1.9–5.9, *p* < 0.001). Lack of awareness (63.2%, 36/57) and lack of time (15.8%, 9/57) were the most common reasons reported for non-receipt of vaccine. Conclusion: Many domestic pilgrims missed the compulsory meningococcal vaccine; in this regard, lack of awareness is a key barrier. Being an overseas pilgrim (or living at a distance from Makkah), receipt of pre-Hajj health advice, and employment were predictors of greater compliance with the vaccination policy. Opportunities remain to reduce the policy–practice gap among domestic pilgrims.

## 1. Introduction

Hajj is a large annual mass gathering that attracts more than two million Muslims from around the world to congregate within confined areas in Makkah, Saudi Arabia. A highly crowded and congested environment during Hajj amplifies risks associated with mass gatherings, including transmission of respiratory organisms, notably *Neisseria meningitidis* [1,2].

*Neisseria meningitidis* is associated with a substantially high rate of carriage (up to 86%) in crowded and closed populations, which resulted in large intercontinental outbreaks of invasive meningococcal disease during Hajj [3]. Following the Hajj in 1987, an intercontinental Hajj-related outbreak of meningococcal serogroup A (MenA) disease led to approximately 2000 cases [4], and its subsequent introduction into the African meningitis belt affected around 70,000 people [5]. Furthermore, in 2000 to 2001, a large outbreak of meningococcal disease resulted in at least 47 deaths, including 11 deaths in the United Kingdom, and affected no fewer than 2400 people in several countries throughout Asia, Africa, Europe, and North America. Serogroup W (MenW; a serogroup that was not previously known to cause large epidemics) sequence type 11 was responsible for over half of those cases [4,6]. 

Mandatory bivalent (serogroups A and C) meningococcal vaccination for all pilgrims from 1987 brought the disease under control during the Hajj for more than a decade [6,7]. Switching the vaccination policy to the quadrivalent (serogroups A, C, W, and Y) meningococcal (MenACWY) polysaccharide vaccine in 2002, coupled with chemoprophylaxis at the port of entry for pilgrims arriving from the African meningitis belt, again brought the subsequent epidemics under control [8]. Since then, no further Hajj-related meningococcal outbreaks occurred [6]. The mandatory vaccination policy also applies to residents of Hajj zones and to personnel who serve pilgrims during the Hajj, including healthcare workers (HCWs) (Table 1) [1,9]. 

Monitoring the annual number of Hajj visas and mandating the vaccine as a prerequisite for the visa application both limited the numbers of overseas pilgrims and improved vaccination rates. For instance, reports on vaccine uptake among overseas pilgrims since 2006 showed a compliance of no less than 96% and reaching up to 100% [10,11,12,13,14]; however, concerns remain among this group of pilgrims, including the receipt of inappropriate vaccines and, due to the limited access to vaccines (including cost), the use of fraudulent vaccination certificates [11,15,16].

Since 2003, Saudi citizens and other expatriate residents in Saudi Arabia who intend to perform Hajj must apply for a Hajj permit with a MenACWY vaccine receipt stipulated as a requirement. Despite this, unauthorized domestic pilgrims often sometimes enter Hajj sites without a permit and without formally registering with an official Hajj tour group. Additionally, despite being enforced and freely offered, the vaccine coverage was found to be very low (64%) in 2006 in the only published work reporting vaccine uptake among domestic pilgrims [12]. The rate was also unsatisfactory among domestic HCWs (ranging from 51.7% to 84.7%) in several studies conducted between 2009 and 2018 [17,18,19,20]. In recent years, the enforcement of the Hajj permit requirement by rigorous procedures at points of entry into Makkah reduced the number and proportion of domestic pilgrims (from 1.4 million (45%) in 2012 to 600,000 (26%) in 2018) [21]. However, there is no recent study assessing the uptake of meningococcal vaccines among these pilgrims. To this end, a survey was undertaken to evaluate the coverage of MenACWY vaccines among Hajj pilgrims and to identify the key predictors and barriers affecting their uptake, particularly among domestic pilgrims, which was not assessed in previous studies. 

## 2. Materials and Methods

An anonymous cross-sectional survey was distributed among domestic pilgrims present in Mina, a tent city, and a main Hajj site on the outskirts of Makkah, and among overseas pilgrims who were staying in Aziziyah (before moving to tents in Mina), a borough of Makkah, adjacent to Mina, during the Hajj seasons of 2017 and 2018. 

### 2.1. Participant Recruitment

Overseas and official domestic pilgrims were eligible to participate; all other non-pilgrims were excluded. In order to recruit a representative sample of both domestic and international pilgrims, the research team approached domestic pilgrims in their camps in Mina, and overseas pilgrims living in hotels/serviced apartments in Aziziyah. The research team (composed of research doctors and trained volunteer allied health or medical students) randomly approached tour operators to access their tent camps or housing and to invite pilgrims to the study. The research team, after obtaining permission from the tour group leaders, explained the study to their pilgrims, answered any queries they had, and invited them to participate. Participation depended primarily on the cooperation of the tour group leader, and then the pilgrim’s willingness to participate.

No identifiable personal data were collected, and respondents’ completion of the survey was considered implied consent. This study was reviewed and approved by the Institutional Review Board of King Saud University College of Medicine, Riyadh, Saudi Arabia (E-17-2534). 

### 2.2. Survey Design

The survey was designed and reviewed by experts in the field of Hajj and vaccine-preventable diseases. The questionnaire collected data on socio-demographic characteristics (such as age, gender, educational level, and employment status), as well as uptake of meningococcal vaccines as a preparation for Hajj and reasons behind non-receipt of the vaccine in such cases. It also evaluated if this was the participant’s first time to the Hajj, whether the vaccine was freely offered, and the receipt of pre-Hajj health advice. The survey was primarily in English, with Arabic translations available for those who preferred to complete the survey in Arabic. Survey responses were collected using a printed or web-based form securely hosted in Wufoo^TM^ (SurveyMonkey Inc., San Mateo, CA, USA). Written responses were entered into the web-based form, and all data were subsequently exported to a Microsoft Excel^TM^ (Microsoft Corp., Redmond, WA, USA) spreadsheet for analysis.

### 2.3. Statistical Methods

The proportion of participants responding to each question was reported. To measure the association between predictors and vaccine uptake, odds ratios (OR) with 95% confidence intervals (95% CI) based on the risk estimate statistics were calculated. Pearson’s chi-squared test was used to compare categorical variables and determine associations and correlations. For questions evaluating sources of pre-Hajj health advice and reasons for non-receipt of the vaccine, one sample nonparametric test (Jeffreys interval) was used to report the proportion of participants providing each response and the 95% CI for the point estimate.

All those who declared previous receipt of the vaccine, regardless of the year of vaccination, were considered as vaccinated; further analysis was done to determine the adherence to the vaccine policy time window. Participants who were unsure about their vaccination history were excluded from the analysis in the OR calculation. A *p*-value ≤ 0.05 was considered statistically significant. The statistical analysis was performed using the Statistical Package for Social Sciences (SPSS^TM^) for Windows^TM^ v.25.0 (IBM Corp., Armonk, NY, USA).

## 3. Results

### 3.1. Participant Characteristics

In total, 513 pilgrims agreed to participate in the study, of whom 509 completed the survey; the remaining four submitted blank forms and, hence, were excluded from the denominator. Only 444 respondents declared their age, ranging from 13 to 82 years (mean 36, SD ±12.6). Males comprised 86% of the sample, and local pilgrims accounted for 85%. Table 2 summarizes the demographic characteristics of the surveyed participants. 

### 3.2. Meningococcal Vaccine Uptake

Of the 476 participants who declared their vaccination status, only 389 (81.7%) confirmed receipt of a meningococcal vaccine; 64 (13.4%), all domestic pilgrims, did not receive the vaccine, and 23 (4.8%) were unsure about their vaccination status. Almost all (93.5% (58/62)) overseas pilgrims declared receipt of the vaccine, although four (6.5%) were unsure, compared with 80.9% (321/397) of domestic pilgrims who received the vaccine (*p* < 0.01), 61/397 (15.3%) who did not, and 15/397 (3.8%) who were unsure (Table 3). Employed participants were twice as likely to be vaccinated as those who were not employed, and those who received pre-Hajj health advice from any source, and those with a tertiary qualification had a higher vaccination uptake rate. Among domestic pilgrims, those from Makkah province were almost three times more likely to miss out on the vaccine compared to those from other provinces.

### 3.3. Participant Adherence to Vaccination Policy

**Figure 1:**
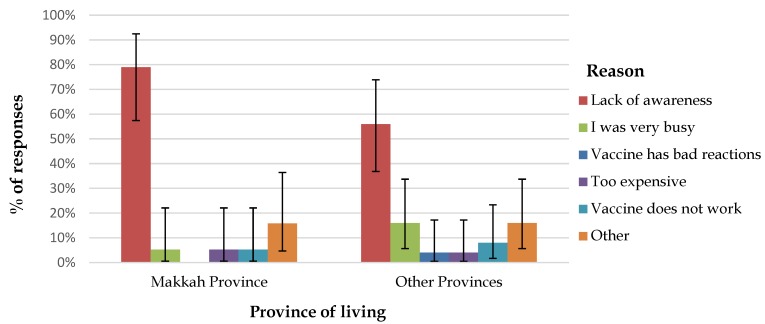
Reasons for non-receipt of meningococcal vaccine among unvaccinated domestic pilgrims: proportion of participants providing each reason with the 95% confidence interval for the point estimate.

Overall, among the 389 vaccinated individuals, 329 (84.6%) received the vaccine within the last three years, 12 (3.1%) received it over three years prior to Hajj attendance, and 48 (11.9%) did not declare the year of vaccination. Thus, 20.5% (70/341) of domestic pilgrims failed to confirm their adherence to the complete vaccination policy (either did not receive the vaccine at all, received it over three years prior, or were unsure about their vaccination status). This translates to an almost seven-fold increased risk of non-compliance with the vaccine policy compared to overseas pilgrims (OR = 6.8, 95% CI = 1.6–28.8), *p* < 0.01). Lack of awareness that the vaccine is a mandatory requirement (63.2%, 36/57) was the main reason given for not receiving the vaccine (Figure 1).

### 3.4. Vaccination Venues

Domestic pilgrims were mainly vaccinated at primary health care centers (79.3%), while overseas pilgrims mostly visited hospitals or travel clinics (70.3%).

### 3.5. Cost of Vaccination

Overall, 55 (15.1%) participants paid for the vaccine. Overseas pilgrims, women, and those who attended Hajj for the first time were significantly more likely to pay for the vaccine than domestic pilgrims, men, or those who attended Hajj previously (Table 4).

### 3.6. Receipt of Pre-Hajj Advice

**Figure 2:**
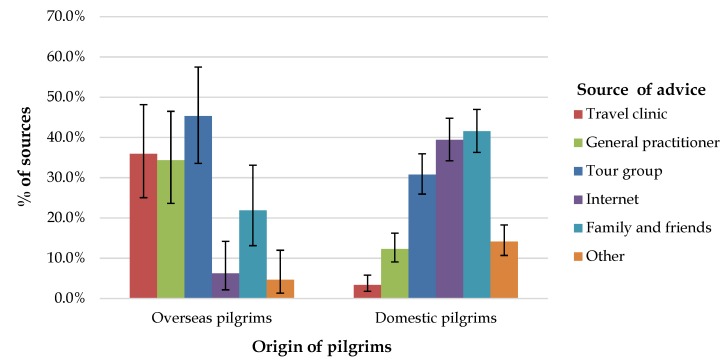
Sources of pre-Hajj health advice among participants who received such advice: proportion of participants providing each response with the 95% confidence interval for the point estimate.

Only 19.4% (98/504) of participants received pre-Hajj health advice from one or more “professional” sources, including general practitioners or a specialized travel clinic; 61% (309/504) received advice from “non-professional” sources, and 19% (97/504) did not receive any advice (Figure 2). Notably, overseas pilgrims (64%) were more likely to receive advice from professional sources than domestic pilgrims (16%; OR = 9.6, 95% CI = 5.3–17.3, *p* < 0.001).

## 4. Discussion

The key finding of this study is that around one-sixth of domestic Hajj pilgrims failed to receive the compulsory MenACWY vaccine in recent years. Meningococcal vaccination is a visa prerequisite for international pilgrims; thus, a high coverage among overseas pilgrims was expected and demonstrated (93.5%). In this regard, the findings of this study are consistent with previous reports. Compliance among overseas pilgrims ranged from 96% to 98% between 2006 and 2010 [13], and two recent studies conducted at King Abdul Aziz International Airport, among 796 and 5235 arriving overseas pilgrims in 2013 and 2014, revealed uptake rates of 98.2% and 100%, respectively [10,11]. However, assessing compliance to other measures of the vaccination policy among overseas pilgrims, such as type and timing of vaccination, is recommended [11,16].

Nevertheless, it is concerning that, despite regulatory efforts, vaccine uptake among local pilgrims who form nearly one-third of total attendees at Hajj each year is unacceptably low. Although the vaccine uptake identified in this survey (85%) is higher than that reported by El Bashir et al. during the Hajj in 2006 (64% among domestic pilgrims who attended the National Guard Clinics in Makkah [12]), it appears that the official regulation that mandates meningococcal vaccination as a prerequisite for a Hajj permit for locals is less effective than that applied to international pilgrims, and a significant number of domestic pilgrims are able to avoid vaccination. Ensuring no Hajj permit is granted unless a valid certificate is provided may improve the situation; however, it is possible that there are more prevailing factors involved, including education of the general population, as well as HCWs. 

Several studies demonstrated suboptimal meningococcal vaccine coverage among local HCWs, which, at best, did not exceed 85% among highly vulnerable hospital emergency room HCWs in Madina in 2015 [19]. A similar rate (82.4%) was also reported among HCWs working in Mina and Arafat, principal Hajj zones in Makkah, in 2003 [17]. Other studies found uptake rates as low as 67.1% and 76.1% among HCWs serving pilgrims in 2009 and 2018, respectively [18,20].

Longer distance of travel appears to act as a motivator for overseas pilgrims to better prepare for Hajj and to seek and follow health advice. This was also noted even among domestic participants in this survey. Similarly, in a previous vaccine uptake survey among domestic pilgrims, fewer pilgrims (50%) from Hajj zones (Makkah and Jeddah) were shown to be vaccinated against MenACWY than pilgrims from other regions in Saudi Arabia (71%) [12]. Moreover, pilgrims from Makkah city were found to have lower vaccination coverage against seasonal influenza than pilgrims from the rest of the country (adjusted OR = 0.52, 95% CI = 0.37–0.72, *p* < 0.001) [22].

An important finding of this survey is that receiving pre-travel health advice, regardless of the source, substantially increased compliance with the vaccination policy. The majority of overseas pilgrims received “professional” pre-Hajj health advice, while locals tended to rely on “social” sources. However, receiving any pre-travel health advice, being employed, and having a tertiary qualification were each individually associated with greater compliance with the vaccination policy. Previous reports on uptake of other recommended vaccines at Hajj also indicated that receiving pre-travel health advice was a considerable motivator for receiving vaccinations against other diseases [14,23]. Furthermore, in a large survey among residents of Gulf Cooperation Council countries, doctors’ advice was the leading motivator for receipt of influenza vaccine [24]. Worksite immunization was shown to be effective in facilitating influenza vaccine uptake in Saudi Arabia [25]. Similarly, some employed participants of this survey indicated receiving the meningococcal vaccine at or through their workplace. This may explain the higher vaccine uptake among employed participants compared with those who identified themselves as unemployed. Additionally, more educated pilgrims were more likely to receive the meningococcal vaccine than those with lower educational attainment. A cross-sectional study of Australian Hajj pilgrims also demonstrated that having a university education was associated with a higher likelihood of receiving recommended Hajj vaccines (OR = 3.4, 95% CI = 1.7–6.7, *p* = 0.01) [14]. Previous reports also described a higher rate of vaccine uptake in women preparing to be pilgrims [20,26,27]. The association observed in this survey was in the same direction, but the difference with men was not statistically significant, which may be due to the low proportion of women who participated in the survey.

Unvaccinated domestic pilgrims named several barriers to vaccination; lack of awareness that the vaccine is compulsory was the most commonly cited reason, followed by lack of time. Lack of awareness as a barrier to vaccination is consistent with pervious findings on meningococcal vaccine uptake during Hajj among local HCWs [19,20]. In fact, lack of knowledge was also highlighted in previous studies reporting uptake of other Hajj recommended vaccines such as influenza vaccine, for both Saudi [22] and international pilgrims (during the influenza A (H1N1) pandemic) [28]. Lack of awareness was also the main reason reported by Australian pilgrims in 2014 for not receiving Hajj recommended vaccines [14]. Lack of time was also found to be a barrier to vaccination among emergency room HCWs in Madina [19] and was shown to be a more significant barrier to influenza vaccination among domestic male pilgrims compared to female pilgrims [22].

Surprisingly, a substantial minority of domestic pilgrims also reported having to pay for their vaccine, which in principle should be provided freely in major public primary healthcare facilities across the country. Unfortunately, the wording of the pre-defined questionnaire had limited ability to identify this as a barrier among domestic pilgrims. 

New, highly immunogenic, conjugate vaccines are replacing the older polysaccharide vaccines in many developed countries, and they are increasingly being recommended for Hajj pilgrims. Conjugate vaccines are more effective in controlling the carriage of meningococci [29,30] but are considerably more expensive. Meningococcal serogroups that are not covered by the current quadrivalent vaccine were frequently isolated from throat swabs collected from pilgrims, namely, serogroups B and, less frequently, X [31,32,33]. A recent systematic review concluded that serogroup B dominated the carriage acquisition among Hajj pilgrims [32], and most carriers received the polysaccharide vaccine, which is not expected to reduce the carriage acquisition of serogroups contained in the vaccine [34,35]. The opportunity to prevent future outbreaks depends on an ongoing review of the current mandatory vaccination policy in view of these and future developments.

Promisingly, most of the participants received some pre-Hajj health advice, but the fact that 84% of vaccinated domestic pilgrims (who certainly had a pre-Hajj contact with health professionals) stated non-receipt of advice from a “professional” source deserves careful attention. This provides an important reminder to local health authorities to take advantage of the national Hajj immunization program as an opportunity for providing face-to-face pre-Hajj health education.

The strength of this survey is that it provides a snapshot regarding the current situation with uptake of the compulsory meningococcal vaccine among mainly domestic Hajj pilgrims, and, for the first time, it provides insight into some of the barriers to vaccination. However, since pilgrims are often too busy to complete forms, the small sample size and the submission of incomplete responses are key limitations of this survey. Additionally, the small number of unvaccinated participants limits the ability to draw reliable conclusions regarding the true role of specific barriers. Furthermore, the data are self-reported, and we had no way of validating vaccination histories; moreover, the questionnaire did not differentiate between conjugate and polysaccharide meningococcal vaccines. Finally, we considered all those who stated previous receipt of the vaccine as vaccinated; however, since some respondents did not state the year of vaccination, the true uptake rates may be lower than reported here. The inability to include “unauthorized” domestic pilgrims also adds to the potential overestimation of the true vaccine uptake rate among domestic pilgrims.

In conclusion, this survey demonstrates that many domestic pilgrims miss the compulsory meningococcal vaccine prior to attending Hajj. Overseas pilgrims appear to have good uptake of the vaccine, as expected from the mandatory vaccination for visa policy. Receipt of pre-travel health advice, regardless of the source, is a key motivator for vaccine uptake, and lack of awareness about the vaccination policy is an important barrier. Improving vaccine uptake likely requires system-wide strategies, such as reducing financial barriers and increasing the availability of vaccination centers, as well as greater education of the public, particularly targeting those who are intending to perform Hajj, regarding Hajj-related health risks and prevention strategies. Strategies to improve the ability of local HCW to proactively provide preventive pre-Hajj health advice are also needed. Additionally, the success of the mandatory vaccination policy that is applied to international pilgrims should be modeled to improve compliance with the domestic policy through more rigorous checks and measures. Ongoing evaluation of such strategies is required to monitor the true uptake of vaccines and other health-promoting behaviors among domestic (and international) pilgrims, so that appropriate public health responses can be made to evolving situations.

## Figures and Tables

**Table 1:** Current preventive measures mandated by the Saudi Arabian government to control meningococcal disease during Hajj.

Measure	When	Age Criteria	Target Group
MenACWY polysaccharide vaccine	Within last 3 years, but ≥ 10 days before arrival	Any individual > 2 years	a. Visitors to Saudi Arabia for Umra *, Hajj, or seasonal work ** b. Residents of Saudi Arabia as follows: - Residents of Makkah and Madina at the time of Hajj - Residents of all other provinces undertaking the Hajj ** - Hajj workers ***
MenACWY conjugate vaccine	Within last 5 years, but ≥ 10 days before arrival		
Chemoprophylaxis (ciprofloxacin, 1 tablet, 500 mg)	Upon arrival (at port of entry)	Any individual > 2 years (excluding pregnant women)	Visitors from African meningitis belt countries for Umra *, Hajj, or seasonal work **

MenACWY; quadrivalent meningococcal serogroup A, C, Y, and W. * A minor pilgrimage to Makkah outside of the Hajj season. ** Requirements for Hajj and Umra entry visa, and for Hajj permit for domestic pilgrims. *** Including individuals working at points of entry or in direct contact with pilgrims.

**Table 2:** Demographic characteristics of the domestic and overseas pilgrims.

Characteristics	All *n*/*N* * (%)	Domestic Pilgrims *n*/*N* * (%)	Overseas Pilgrims *n*/*N* * (%)	*p*-Value
**Number of participants**	509^**^	416/489 (85.1)	73/489 (14.9)	
**Age in years**				
Mean (SD)	36 (± 12.6)	34.7 (± 12)	42 (± 13.4)	< 0.001 ***
Range (Median)	13–82 (33.8)	13–82 (32.6)	21–69 (38.9)	
**Gender**				
Male:female	6:1	7.5:1	3:1	0.03 ***
**Country of residence**				
Saudi Arabia	398/499 (79.8)	398/412 (96.6)	0	
Pakistan	33/499 (6.6)	0	30/73 (41.1)	
South Africa	28/499 (5.6)	0	28/73 (38.4)	
Egypt	11/499 (2.2)	7/412 (1.7) ^¶^	0	
Malawi	6/499 (1.2)	0	6/73 (8.2)	
Zambia	5/499 (1)	0	5/73 (6.8)	
Other	18/499 (3.6)	7/412 (1.7) ^¶^	4/73 (5.5)	
**Highest qualification**				
No formal education	4/499 (0.8)	2/411 (0.5)	1/70 (1.4)	
School certificate ^ǂ^	43/499 (8.6)	34/411 (8.2)	4/70 (5.7)	
High school certificate ^§^	124/499 (24.8)	100/411 (24.3)	21/70 (30)	
Diploma	58/499 (11.6)	39/411 (9.5)	12/70 (17.1)	
University undergraduate degree	215/499 (43.1)	184/411 (44.8)	30/70 (42.9)	
University postgraduate degree	55/499 (11)	52/411 (12.7)	2/70 (2.9)	
**Employed**				
No	168/501 (33.5)	134/412 (32.5)	25/69 (36.2)	0.61
Yes	333/501 (66.5)	278/412 (67.5)	44/69 (63.8)	
**Hajj attendance**				
First time	285/500 (57)	212/409 (51.8)	60/71 (84.5)	< 0.001 ***
> 1 time previously	215/500 (43)	197/409 (48.2)	11/71 (15.5)	

SD—standard deviation. * The total number of respondents with complete information for each individual variable. ** Twenty participants with unknown allocation status (overseas or domestic). *** Statistically significant. ^¶^ Holders of any visa other than a Hajj visa were officially treated as domestic pilgrims. ^ǂ^ Year 10 equivalent; ^§^ year 12 equivalent.

**Table 3:** Meningococcal vaccine uptake.

Characteristics	Overall Meningococcal Vaccine Uptake			Meningococcal Vaccine Uptake among Domestic Pilgrims		
	*n*/*N* * (%)	OR ** (95% CI)	*p*	*n*/*N* * (%)	OR ** (95% CI)	*p*
**All**						
All participants ^¶^	389/476 (81.7)			321/397 (80.9)		
**Gender**						
Male	322/380 (84.7)	1.0 (ref)		270/326 (82.8)	1.0 (ref)	
Female	53/58 (91.4)	1.9 (0.7–4.98)	0.18	39/43 (90.7)	2 (0.7–5.9)	0.19
**Origin**						
Domestic	321/382 (84)	1.0 (ref)				
Overseas	58/58 (100)	n.a	<0.001 ^§^			
**Province of residence**						
Makkah Province				59/80 (73.8)	1.0 (ref)	
Other				207/235 (88.1)	2.6 (1.4–5)	< 0.01 ^§^
**Hajj attendance**						
First time	208/246 (84.6)	1.0 (ref)		158/193 (81.9)	1.0 (ref)	
≥ 1 time previously	174/199 (87.4)	1.3 (0.7–2.2)	0.39	158/183 (86.3)	1.4 (0.8–2.4)	0.24
**Tertiary qualification**						
No	122/150 (81.3)	1.0 (ref)		99/125 (79.2)	1.0 (ref)	
Yes	262/297 (88.2)	1.7 (1.0–2.5)	0.048 ^§^	220/254 (86.6)	1.7 (0.97–2.98)	0.06
**Employed**						
No	113/144 (78.5)	1.0 (ref)		92/121 (76)	1.0 (ref)	
Yes	269/302 (89.1)	2.2 (1.3–3.8)	0.03^§^	226/258 (87.6)	2.2 (1.3–3.9)	< 0.01 ^§^
**Received pre-Hajj health advice**						
No	64/89 (71.9)	1.0 (ref)		54/78 (69.2)	1.0 (ref)	
Yes	322/360 (89.4)	3.3 (1.9–5.9)	< 0.001 ^§^	264/300 (88)	3.3 (1.8–5.9)	< 0.001 ^§^

OR, odds ratio; 95% CI, 95% confidence interval; *p*, *p*-value; ref, reference value; n.a, not available. * Total number of respondents with vaccination status (excluding “unsure” respondents) and complete information for each individual variable. ** For OR calculation, responses with “unsure” for vaccination status were excluded. ^¶^ Includes all participants with vaccination status. ^§^ Statistically significant.

**Table 4:** Covering the cost of vaccination.

Characteristics		Proportion of Participants Who Paid for the Vaccine		
		*n*/*N* * (%)	OR (95% CI)	*p*
**All**				
All participants ^¶^		55/364 (15.1)		
**Gender**				
Male		38/302 (12.6)	1.0 (ref)	
Female		16/49 (32.7)	3.4 (1.7–6.7)	< 0.001 ^§^
**Origin**				
Domestic		30/307 (9.8)	1.0 (ref)	
Overseas		23/48 (47.9)	8.5 (4.3–16.7)	< 0.001 ^§^
	Pakistan	17/17 (100)		
	South Africa	3/20 (15)		
	Other	9/27 (33.3)		
**Hajj attendance**				
≥ 1 time previously		13/165 (7.9)	1.0 (ref)	
First time		41/193 (21.2)	3.2 (1.6–6.1)	< 0.001 ^§^
**Tertiary qualification**				
Yes		31/246 (12.6)	1.0 (ref)	
No		22/112 (19.6)	1.7 (0.9–3.1)	0.09
**Employed**				
Yes		32/246 (13)	1.0 (ref)	
No		21/108 (19.4)	1.6 (0.9–3)	0.12

OR, odds ratio; 95% CI, 95% confidence interval; *p*, *p*-value; ref, reference value. * Total number of respondents with known source of payment and complete information for each individual variable. ^¶^ Includes all participants with known source of payment. ^§^ Statistically significant.
